# Socio-demographic and clinical predictors of medication adherence among psychiatric outpatients in Mozambique: A two-year longitudinal survival analysis

**DOI:** 10.1371/journal.pmen.0000236

**Published:** 2025-02-03

**Authors:** Hiwot Weldemariam, Morgan Turner, Alberto Gabriel Muanido, Vasco Cumbe, João Jala Junior, Ernesto Eusébio Armando, Flávia Faduque, Daniel A. Enquobahrie, Bradley H. Wagenaar

**Affiliations:** 1 Department of Epidemiology, University of Washington, Seattle, Washington, United States of America; 2 Department of Global Health, University of Washington, Seattle, Washington, United States of America; 3 Mozambican Health Committee, Beira, Mozambique; 4 Centre of Training and Research, Beira Central Hospital, Beira, Sofala, Mozambique; 5 Provincial Health Directorate, Manica Province, Ministry of Health, Chimoio, Mozambique; West Virginia University, UNITED STATES OF AMERICA

## Abstract

Research on medication adherence among patients with psychiatric illness is particularly sparse in low- and middle-income countries (LMICs). This study examined the relationship between psychiatric medication adherence and clinical and sociodemographic factors in outpatient psychiatric settings in central Mozambique. We conducted a longitudinal study among 803 Psychiatric Outpatients (1,811 total follow-up visits) attending eight healthcare facilities in Mozambique from February 2022 to January 2024. Multivariable linear mixed and non-parametric Kaplan-Meier models were employed to analyze the association between medication and clinical/sociodemographic variables over time. Approximately 93% of patients (N = 729) were non-adherent to their medication at a minimum of one follow-up visit, with a median time in treatment prior to non-adherence of 60 days (mean: 52 days; 95% CI: 51, 53). In the Stratified Cox model, patients aged 56+ had a 33% higher hazard of nonadherence compared to those aged 18–35 (aHR: 1.33; 95% CI: 1.14, 1.55). Patients prescribed amitriptyline had a 56% higher hazard of nonadherence compared to those prescribed carbamazepine (aHR: 1.56; 95% CI: 1.23, 1.98). For every 30 days in treatment, disability scores decreased by an average of 0.13 (95% CI: -0.16 to -0.09) while systolic blood pressure decreased by 0.46 mmHg (95% CI: -0.18 to -0.10) and diastolic blood pressure decreased by 0.15 mmHg (95% CI: -0.23 to -0.06). Currently, the median duration of medication adherence for patients initiating essential psychiatric treatment in Mozambique is 60 days. Patients prescribed amitriptyline and older patients are at a higher risk of non-adherence. Consistent engagement in treatment is linked to lower disability scores and blood pressure. There is an urgent need for research into adherence support strategies, especially for these high-risk groups within Mozambique’s mental health patient population.

## Introduction

The World Health Organization (WHO) defines mental, neurological, and substance use disorders (MNS) as disruptions in thought, emotions, and psychosocial functioning, influenced by biological, psychological, and social factors [1]. MNS are leading causes of disability, premature mortality [2], high economic costs [direct and indirect] due to lost work time [3], and reduced productivity [4]. According to 2020 data, MNS disorders account for 14% of the global burden of disease (GBD). This indicates an increase from previous years, highlighting the growing impact of these disorders worldwide [1]. This burden is particularly significant in low- and middle-income countries (LMICs), where 75% of those affected do not receive the treatment they need [5]. Across all age groups, schizophrenia, depression, epilepsy, dementia, alcohol dependence, and other mental, neurological, and substance use disorders constitute 13% of the GBD and account for 10.4% of disability-adjusted life years globally in 2010 [6]. In addition, patients with mental disorders are stigmatized [7], making it a hidden challenge for help-seeking. Societal issues such as armed conflict, post-conflict situations, and poverty, common in Sub-Saharan Africa, are also known to be detrimental to mental health and care-seeking [8].

Across Mozambique, a country with a population of 33.8 million as of 2024 [9], over 23.9% of patients attending primary care may have common mental disorders [10]. The prevention, care, and treatment of mental disorders have been historically neglected compared with other health conditions [11]. Despite efforts by the Ministry of Health to close the treatment gap through a task-shifting approach, systematic barriers such as lack of resources, supervision issues, and medication stock-outs contribute to the poor quality of mental health care.

Nonadherence to treatment poses a significant challenge among patients with psychiatric illness, with reported rates ranging from 20% to 60% globally [12]. Nonadherence can present itself in several ways, such as not taking medications as prescribed [wrong dosage or frequency], stopping the medication entirely, attending follow-up appointments irregularly, or, in some cases, being fully lost to treatment follow-up. The rate of nonadherence to typical antipsychotics is notably higher in Sub-Saharan Africa compared to other continents, with figures ranging from 46.9% to 93.3%, in contrast to 11% in China and 25.6% in Bulgaria [13]. Furthermore, the problem of inadequate adherence to medical treatment is expected to grow due to limited and uneven access to healthcare services and medications as treatment continues to be scaled up in developing nations [14].

Poor adherence is a multifactorial phenomenon that can result from five major interacting factors: the health team and health system, the patient, therapy, social/economic factors, and clinical factors [15]. Medication non-adherence can undermine mental healthcare effectiveness, resulting in poor health outcomes and increased costs. These consequences include frequent hospital admissions, relapses or recurring symptoms, a heightened risk of suicide or dependency, reduced functionality, increased absenteeism, rebound effects, comorbidities such as weight gain and cardiometabolic disorders, and higher mortality rates [15–17].

A recent pilot study conducted in Mozambique revealed that mental health patients were adherent to medication in only 17% of follow-up visits and showed improvement in daily living activities at 26.6% of these visits [18]. However, this study did not investigate the factors influencing medication adherence or the potential effects of mental health treatment on patients’ physical health and disability scores over time.

Research on adherence and related factors, as well as adherence support interventions, is limited across sub-Saharan Africa, and no studies have yet been published in Mozambique. The primary objective of this study was to determine the socio-demographic and clinical predictors of medication non-adherence among this cohort. The secondary objective was to examine the relationship between the primary medications prescribed to these patients and changes in their World Health Organization Disability Assessment Schedule 2.0 (WHODAS-MZ) scores, weight, and blood pressure. The findings will offer valuable insights into the factors driving medication non-adherence and its biological implications, providing an evidence base to guide and inform strategies for addressing this widespread challenge among patients with psychiatric illness in Mozambique and other LMICs more broadly.

## Materials and methods

### Study design and study setting

We conducted a secondary data analysis using cohort data collected as part of the System Analysis and Improvement Approach for Mental Health (SAIA-MH) cluster randomized controlled trial [19]. This trial employed a cluster-randomized design across 16 clinics (eight intervention and eight control) providing primary mental health care in Sofala and Manica provinces of Mozambique. Detailed information on health facility eligibility and selection is available in the published trial protocol [19]. The current secondary analysis of trial data uses data from control facilities. The trial protocol was reviewed and approved by the University of Washington IRB and the National Ethics Committee in Mozambique (CNBS).

In Mozambique, the Ministry of Health oversees public hospitals and healthcare centers, providing services to the majority of the population [20]. Patients are required to visit a public medical clinic to obtain a formally signed, dated, and stamped prescription, which allows them to purchase medications from the hospital’s pharmacy for a state-subsidized fee of 5 Meticais (< USD 0.1). If the medication is unavailable locally, they may purchase from private pharmacies—which generally do not require a prescription—although psychiatric medications at private pharmacies cost significantly more. While treatments are state-subsidized, availability can be irregular.

### Study population and data source

The current study utilized data from 803 Psychiatric Outpatients and their 1,811 routine follow-up visits with psychiatric technicians between February 2022 and January 2024 at eight primary healthcare facilities in the Sofala and Manica provinces of Mozambique. This study included a census of all patients who were either new to the treatment or who had not been seen at the facilities for psychiatric treatment for at least six months at the initiation of the study baseline period of February 2022. In our study, **"patients with psychiatric illness"** include all individuals receiving care at the psychiatric department, encompassing both mental and neurological disorders. In Mozambique, the healthcare infrastructure and resource allocation often result in patients with neurological conditions like epilepsy being managed within psychiatric services. These patients completed WHODAS disability screening on their first visit and received care from psychiatric technicians for mental health conditions that required medication. Patients who were being treated solely for medical conditions unrelated to mental health were excluded.

### Data collection

During the provision of routine primary mental healthcare, psychiatric technicians collected clinical data from patients using enhanced paper-based registries and patient charts. These newly introduced registries and charts facilitated the systematic tracking of patient visits, medications dispensed, and the progression of disability over time (using the WHODAS). To ensure this information was efficiently managed and updated, research assistants digitized the patient data monthly using the CommCare application. This process of data collection and digitization allowed for a comprehensive and accurate compilation of clinical data, essential for the detailed analysis and assessment of patient outcomes within the study.

### Exposures

The primary exposure variables in this study include primary diagnoses (epilepsy, schizophrenia-related disorders, other psychotic and delusional disorders, depression-related disorders, mental and behavioral disorders due to substance use, and all other diagnoses). (see **Table A in S1 Text**). "All other diagnoses" are grouped with any ICD-10 code [21] psychiatric diagnoses with fewer than 30 patients each (see **Table B in S1 Text**). Primary medications (Carbamazepine, Haloperidol, Amitriptyline, Thioridazine, and all other medications). (see **Table C in S1 Text)**. "All other medications," grouped those medications with fewer than 30 patients each (see **Table D in S1 Text**). Sociodemographic characteristics included age (<18, 18–35, 36–55, and over 56 years), sex (female and male), and marital status (single, married, divorced, separated, widowed, and common-law).

### Outcomes

Our primary outcome of interest was adherence to treatment. This metric was determined through a dual-question approach. The first question involved directly asking the patient if they adhered to the prescribed medication (i.e., taking the treatment as prescribed). The second aspect involved cross-referencing the patient record to confirm whether the patient attended the follow-up appointment before exhausting their medication supply. In the Mozambican health system, patients must return for follow-up visits to receive their routine medications. The patient needed to fulfill these two conditions to be considered adherent to the treatment at every visit.

The secondary outcomes of our study included weight (measured in kilograms), systolic and diastolic blood pressures (measured in mmHg), and the WHODAS score. The WHODAS score is a tool used to measure health and disability across six domains of functioning: cognition, mobility, self-care, social interactions, life activities, and societal participation. We used WHODAS 2.0 with a maximum possible score of 48. Higher scores indicate greater levels of disability. WHODAS is designed to be universally applicable across different cultures and adult populations [22]. As part of routine clinical delivery, these variables are measured at each visit per protocol.

### Statistical analysis

We used descriptive statistics to summarize baseline characteristics of the study population, using frequencies and percentages for categorical and dichotomous variables and mean and standard deviation (SD) for continuous variables. We conducted survival analysis—a statistical method designed to model time-to-event [23] data—to examine factors related to medication non-adherence.

We employed a non-parametric Kaplan-Meier model to estimate survival (time to non-adherence) across various exposure variables. To account for the clustering of patients within each facility, the model was adjusted accordingly. The differences in survival among each category of exposure variables were compared using the log-rank test. To determine which baseline factors influenced the time to non-adherence, we utilized a multivariate Cox Proportional Hazards (CPH) model. Additionally, a stratified Cox model was considered when the proportional hazards assumption was violated [24]. The CPH model assumes that the hazard ratio for any two specifications of predictors remains constant over time. Schoenfeld residuals can be used to assess whether the Cox Proportional Hazard (PH) assumption holds. The “Stratified Cox (SC)” model is a modification of the CPH model that allows for the stratification of a predictor that does not meet the PH assumption. Predictors that violate the PH assumption are adjusted by stratification, while predictors that satisfy the assumption are included in the model and adjusted accordingly. The hazard ratios for variables that satisfy the assumption can be estimated within each stratum; however, hazard ratios for the stratified variable itself cannot be estimated [24–26]. Non-adherence was defined as the event of interest, and ’time’ was measured as the number of days for which medications were supplied until the first occurrence of non-adherence. For analysis purposes, patients were categorized into three groups based on their follow-up visits: The first group consisted of 240 patients who attended the clinic only once and did not return. For these individuals, the time to event was assumed to be the duration of their initial medication supply. The second group, comprising 489 patients with more than one visit, was followed until their first non-adherence event. The time to event for this group was calculated as the total duration of medication supplied during the observation period. The third group included 57 patients who remained adherent throughout the study. For these patients, the last adherent visit and the cumulative duration of medication supply until that point were used to determine the time to event. Right censoring was applied to these 57 patients, who were consistently adherent until the end of the study period. There were also 17 patients who were left censored due to a lack of complete data on medication duration. We reported the hazard of failing to adhere to the first medication using the adjusted hazard ratio (aHR), along with 95% confidence intervals (CI) and p-values. The adjusted models included variables for primary diagnoses, primary medication, age, sex, and marital status. For each variable category, Schoenfeld residuals were used to assess the proportional hazards (PH) assumption. Based on the results, either Cox’s PH model or a stratified Cox model was applied, depending on whether the PH assumption was satisfied.

A multivariable generalized linear mixed model [27] was employed to examine the associations of primary medications, primary diagnosis, age, sex, marital status, and the duration of 30 days in treatment with changes in the outcome variables: weight, blood pressure, and WHODAS score. These models incorporated a patient-level random intercept to account for clustering. We reported the adjusted estimates (β) with their 95% CI. All tests were 2-sided and p < 0.05 was used to denote statistical significance. All analyses were conducted using R statistical software, version 4.3.1.

### Ethics

This study involved no new human subjects research or collection of identifiable data, as it was conducted as a secondary data analysis using de-identified data from the SAIA-MH cluster randomized controlled trial. The original trial protocol was reviewed and approved by the University of Washington Institutional Review Board (IRB) and the National Ethics Committee in Mozambique (CNBS).

## Results

Most study participants were aged 18–35 years (60%), with a mean age of 31 years. Around half were male (53%), single (63%), and had a normal body mass index (BMI, 18.5–24.9 kg/m^2^) with 62% falling into this category and an average BMI of 19 kg/m^2^. Sixteen percent of participants were HIV positive, 18% were current alcohol users, and 9% were current drug users. At enrollment, 3% of participants reported thoughts of suicide. The mean WHODAS score was 11, with a mean systolic blood pressure of 95 mmHg, a mean diastolic blood pressure of 78 mmHg, and a mean weight of 49 kg. (see **Table 1**).

**Table 1:** Selected socio-demographic characteristics of psychiatric outpatients in Mozambique from February 2022 to January 2024.

Patient Characteristics	Total N = 803*
Age [Mean/SD] in years	31 [14]
<18	77 [9.80%]
18–35	479 [59.81%]
36–55	174 [21.20%]
56+	73 [9.21%]
Sex	
Female	378 [47.10%]
Marital status	
Married	61 [7.60%]
Divorced	6 [0.80%]
Separated	26 [3.21%]
Single	507 [63.10%]
Widowed	45 [5.61%]
Common-law	155 [19.30%]
WHODAS Score [Mean/SD]	11 [7]
0–9	348 [43.30%]
9–20	389 [48.41%]
20–30	59 [7.42%]
30+	7 [0.90%]
Weight [Mean/SD] Kg	49 [23]
Systolic blood pressure [Mean/SD] mmHg	95 [49]
<120	269 [33.53%]
120–129	276 [34.42%]
130–139	79 [9.90%]
140+	53 [6.60%]
Missing	126 [15.60%]
Diastolic blood pressure [Mean/SD] mmHg	78 [11]
<80	317 [39.50%]
80–89	271 [33.60%]
90+	90 [11.21%]
Missing	125 [15.71%]
BMI [Mean/SD] kg/m2	19 [10]
<18.5	103 [12.80%]
18.5–24.9	501 [62.41%]
25–29.9	77 [9.62%]
30+	25 [3.10%]
Missing	97 [12.11%]
HIV Positive Status	
Yes	127 [15.82%]
No	462 [57.52%]
Unknown	208 [25.90%]
Missing	6 [0.80%]
TB Positive status	
Yes	32 [4.00%]
No	361 [45.01%]
Unknown	399 [49.71%]
Missing	11 [1.32%]
Alcohol use status	
Current user	144 [17.91%]
Never user	494 [61.52%]
Past user	134 [16.73%]
Missing	31 [3.91%]
Drug use status	
Current user	69[8.61%]
Never user	454 [81.40%]
Past user	65 [8.11%]
Missing	15 [1.91%]
Thoughts of suicide	
No	775 [96.50%]
Yes	24 [3.01%]
Missing	4 [0.50%]
Patient pregnancy status	
No	794 [98.80%]
Yes	6 [0.81%]
Missing	3 [0.41%]
Patient engaged in family planning	
No	768 [95.60%]
Yes	32 [4.00%]
Missing	3 [0.40%]

Approximately 93% (729 out of 786) of patients failed to adhere to their primary medication at least once during the study period. In our non-parametric Kaplan-Meier analysis, after adjusting for facility clustering, the median survival time before non-adherence was 60 days, and the mean survival time was 52 days (95% CI: 51, 53). There was no significant difference in median survival time between male and female patients, both being 60 days (p-value = 0.70 from the log-rank test for sex-specific differences). In age-stratified groups, median survival times ranged from 52.5 days for individuals under 18 years old to 60 days for those aged 56 and older (p-value for age-specific differences = 0.10). The median survival time was similar across marital status groups (60 days), except for individuals who were divorced, with a median survival time of 33.5 days (p-value for differences across marital status groups = 0.20).

**Fig 1:**
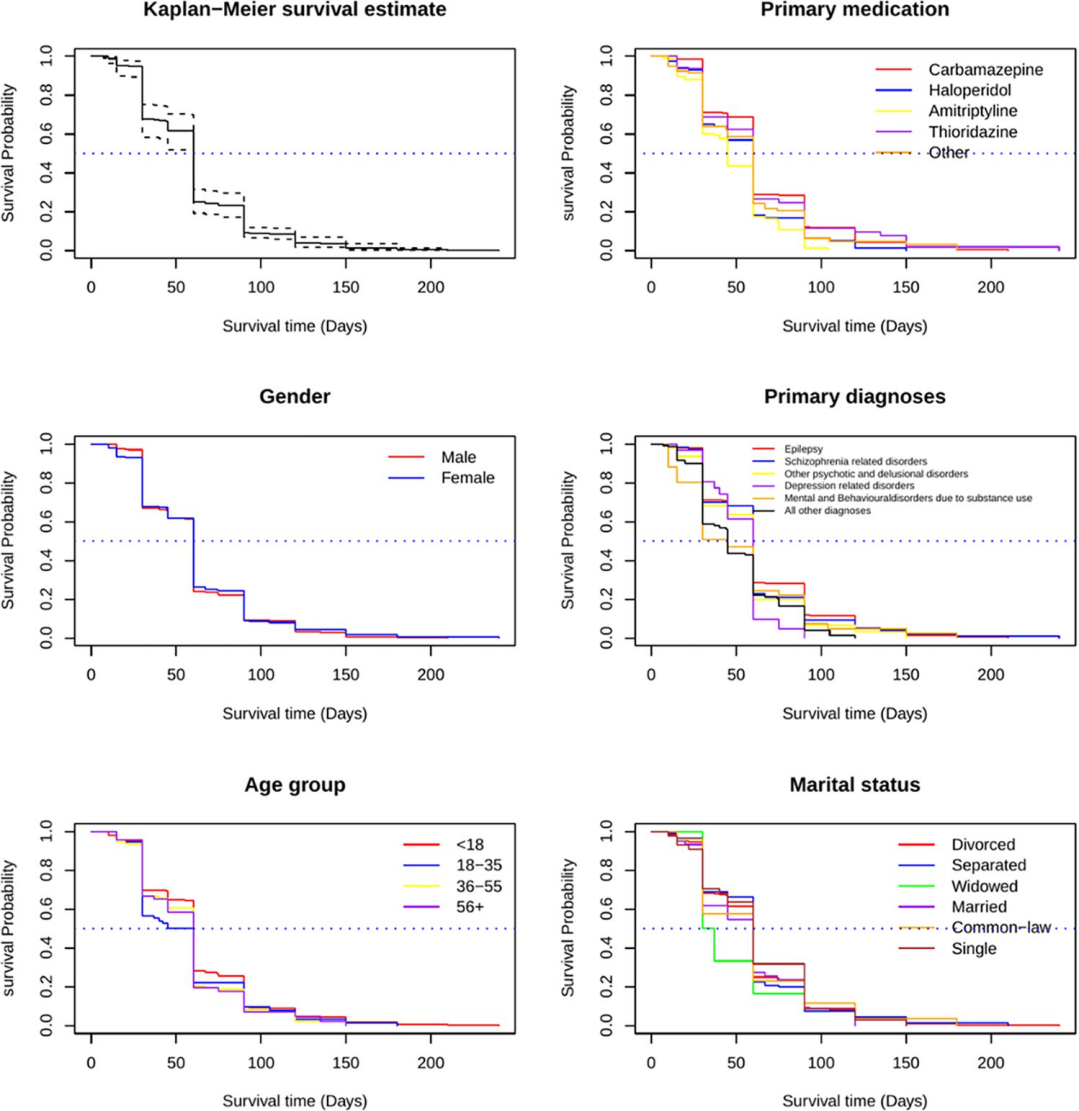
Kaplan–Meier survival curves (overall and stratified by sociodemographic characteristics) of non-adherence to the primary medication among psychiatric outpatients in Mozambique (February 2022–January 2024).

For patients with mental and behavioral disorders due to substance use and those in the “all other diagnosis” group, the median survival time was 45 days (see **Fig 1**). In contrast, patients with epilepsy, schizophrenia-related disorders, other psychotic and delusional disorders, and depression-related disorders had a median survival time of 60 days (p-value for differences across diagnoses < 0.001). Patients taking Amitriptyline had a median survival time of 45 days, while those taking carbamazepine, haloperidol, thioridazine, and the “other medication” group had a median survival time of 60 days (p-value for differences across medications < 0.001).

The results of testing the proportional hazards assumption are presented in Supplementary Table E in S1 Text. Based on the chi-square values, the only variable that did not satisfy the proportional hazards assumption was "primary diagnoses." In the stratified Cox model, individuals aged 56+ had a 33% higher hazard of non-adherence compared with those aged 18–35 (aHR: 1.33; 95% CI: 1.14, 1.55) (see **Table 2**). Patients prescribed amitriptyline had a 56% higher hazard of non-adherence compared to those prescribed carbamazepine (aHR: 1.54; 95% CI: 1.23, 1.98). Additionally, patients in the ‘all other medications’ group had a 19% higher hazard compared to those prescribed Carbamazepine (aHR: 1.19; 95% CI: 1.01, 1.40). Gender and marital status were not associated with nonadherence.

**Table 2:** Factors associated with medication non-adherence among psychiatric outpatients in Sofala and Manica provinces, Mozambique [February 2022 –January 2024] post-diagnostic test and stratified Cox proportional hazards model.

Variables	Nonadherence N [%] [95% CI]**	Crude model		Stratified model^	
		HR	95% CI	aHR*	95% CI
Sex: Male	383 [93%] [90%, 94%]	0.96	[0.83, 1.13]	0.97	[0.80, 1.19]
**Age [Years]**					
*18–35 [ref]*	434 [92%] [89%, 94%]				
*<18*	74 [96%] [88%, 99%]	1.20	[0.83, 1.73]	1.22	[0.81, 1.85]
*36–55*	153 [92%] [86%, 95%]	1.18	[1.04, 1.34]**	1.14	[0.93, 1.40]
*56+*	68 [94%] [85%, 98%]	1.20	[0.84, 1.70]	1.33	[1.14, 1.55]**
**Marital Status**					
*Single [ref]*	461 [93%] [90%, 94%]				
*Married*	55 [92%] [82%, 97%]	1.05	[0.77, 1.45]	1.01	[0.68, 1.53]
*Separated*	26 [100%] [86%, 100%]	1.00	[0.52, 1.91]	0.99	[0.55, 1.81]
*Divorced*	5 [83%] [36%, 99%]	1.17	[0.34, 3.95]	0.72	[0.19, 2.82]
*Widowed*	40 [91%] [77%, 97%]	0.94	[0.70, 1.25]	0.74	[0.53, 1.04]
*Common Law*	139 [935] [87%, 96%]	1.01	[0.87, 1.17]	0.93	[0.77, 1.12]
**Primary medication**					
*Carbamazepine [ref]*	366 [92%] [89%, 94%]				
*Haloperidol*	83 [97%] [90%, 99%]	1.37	[0.85, 2.20]	1.34	[0.97, 1.87]
*Amitriptyline*	108 [93%] [86%, 96%]	1.70	[1.28, 2.26]***	1.56	[1.23, 1.98]**
*Thioridazine*	61 [95%] [86%, 98%]	1.01	[0.73, 1.41]	0.95	[0.60, 1.51]
*All other medications ℸ*	109 [91%] [84%, 95%]	1.21	[1.10, 1.33]***	1.19	[1.01, 1.40]**
**Primary Diagnosis**					
*Epilepsy [ref]*	372 [92%] [89%, 94%]				
*Schizophrenia related disorders*	206 [96%] [92%, 98%]	1.07	[0.94, 1.22]		
*Other psychotic and delusional disorders*	43 [91%] [78%, 97%]	1.23	[0.90, 1.69]		
*Depression related disorders*	30 [97%] [83%, 99%]	1.54	[1.28, 1.85]***		
*Mental and Behavioral disorders due to substance use*	48 [94%] [83%, 98%]	1.33	[0.77, 2.30]		
*All other “rare” diagnoses ∂*	130 [92%] [86%, 95%]	1.51	[1.22, 1.87]***		

Significant codes: 0.01 ‘***’ 0.05 ‘**’

^Stratified Cox Proportional Hazards Mode (adjusted)

* aHR—adjusted hazard ratio

** Categories with small sample sizes the confidence intervals are wider due to greater variability

ℸ All other medications (see Table D **in S1 Text**)

∂ All other diagnoses (see Table B **in S1 Text**)

In fully-adjusted generalized linear mixed models, for each 30 days in treatment, WHODAS score, systolic blood pressure, and diastolic blood pressure all showed significant reductions over time, with an average reduction of 0.13 (95% CI: -0.16, -0.09), 0.46 mmHg (95% CI: -0.81, -0.10), and 0.15 mmHg (95% CI: -0.23, -0.06), respectively (see **Table 3**). Across all visits, patients diagnosed with schizophrenia-related disorders and patients in the ‘all other diagnoses group’ had 2.0 (95% CI: 0.88, 3.17) and 1.8 (0.69, 2.91) higher disability scores, respectively, compared with patients diagnosed with epilepsy. During the study period, male patients weighed 3.32 kg more than female patients (95% CI: 0.63 to 6.02). Additionally, males had marginally lower diastolic blood pressure (DBP) compared to females, with a difference of 4.39 mmHg (95% CI: -8.51,-0.27). Patients in the ’all other medication’ group had a weight that was 3.21 kg lower (95% CI: -6.41, 0.01) compared to those taking carbamazepine.

**Table 3:** Associations of biological variables [WHODAS score, SBP, DBP, and weight] with primary medication and diagnosis among psychiatric outpatients in Sofala and Manica province, Mozambique from February 2022 to January 2024.

Predictor variables	WHODAS score			SBP (mmHg)			DBP (mmHg)			Weight (kg)		
	aβ*	SE(β)	95% CI	a*β*	SE(β)	95% CI	a*β*	SE(β)	95% CI	a*β*	SE(β)	95% CI
**Intercept**	9.68	1.69	[6.35, 13.02]***	98.22	4.54	[89.32, 117.12]***	81.63	2.3	[77.51, 85.75]***	48.9	1.68	[45.59, 52.21]***
**Days [30 days]**	-0.13	0.02	[-0.16, -0.09]***	-0.46	0.18	[-0.81, -0.10]**	-0.15	0.04	[-0.23, -0.06]***	-0.05	0.08	[-0.20, 0.12]
**Sex: Male**	-0.34	0.39	[-1.11, 0.42]	2.22	2.61	[-2.90, 7.34]	-4.39	2.10	[-8.51, -0.27]**	3.32	1.37	[0.63, 6.02] **
**Age [Years]**												
*18–35 [ref]*												
*36–55*	-0.3	0.47	[-1.22, 0.63]	0.01	3.15	[-6.19, 6.21]	2.48	2.54	[-2.50, 7.47]	0.88	1.66	[-2.38, 4.14]
*56+*	0.65	0.67	[-0.67, 1.97]	2.33	4.47	[-6.44, 11.10]	3.43	3.60	[-3.63, 10.50]	-2.88	2.35	[-7.49, 1.74]
**Marital status**												
*Single [ref*]												
*Married*	0.08	0.74	[-1.37, 1.54]	-3.8	4.98	[-13.72, 6.12]	-3.21	3.96	[-11.07, 4.63]	-0.88	2.58	[-6.02, 4.26]
*Separated*	0.92	1.03	[-1.11, 2.94]	6.17	6.71	[-7.05, 19.39]	-3.00	5.26	[-13.33, 7.33]	4.98	3.53	[-1.98, 11.94]
*Divorced*	-1.46	2.1	[-5.59, 2.67]	17.09	14.86	[-12.21, 46.38]	-10.46	10.73	[-31.52, 10.61]	0.24	7.65	[-14.84, 15.32]
*Widowed*	-0.06	0.49	[-1.02, 0.90]	2.56	3.24	[-3.80, 8.92]	-1.41	2.52	[-6.36, 3.55]	3.33	1.61	[-0.90, 6.02]
**Primary medication**												
*Carbamazepine [ref]*												
*Haloperidol*	0.62	0.57	[-0.50, 1.75]	-3.97	5.08	[-13.94, 5.99]	-0.93	1.63	[-4.13, 2.27]	-3.57	2.45	[-8.38, 1.22]
*Amitriptyline*	-0.65	0.61	[-1.86, 0.53]	8.49	5.16	[-1.64, 18.61]	-0.76	1.9	[-4.49, 2.96]	2.14	2.52	[-2.79, 7.09]
*Thioridazine*	-0.57	0.49	[-1.55, 0.40]	1.18	4.76	[-8.15, 10.51]	-0.5	1.35	[-3.15, 2.15]	-1.71	2.26	[-6.15, 2.72]
*All others ℸ*	0.48	0.36	[-0.22, 1.18]	-3.34	3.46	[-10.12, 3.44]	0.97	0.99	[-0.97, 2.90]	-3.21	1.63	[-6.41, 0.01]**
**Primary diagnosis**												
*Epilepsy [ref]*												
*Schizophrenia related disorders*	2.02	0.58	[0.88, 3.17]***	3.39	4.75	[-5.93, 12.72]	-2.74	1.99	[-6.63, 1.17]	0.36	2.36	[-4.28, 5.00]
*Other psychotic and delusional disorders*	-0.51	0.69	[-1.86, 0.86]	8.07	5.62	[-2.96, 19.10]	-0.61	2.36	[-5.24, 4.02]	5.04	2.81	[-0.47, 10.57]
*Depression related disorders*	0.73	0.92	[-1.07, 2.53]	9.37	7.52	[-5.37, 24.12]	3.31	2.95	[-2.47, 9.09]	2.43	3.75	[-4.92, 9.78]
*Mental and Behavioral disorders due to substance use*	1.42	0.8	[-0.16, 2.99]	2.1	6.23	[-10.11, 14.31]	3.48	3.38	[-3.14, 10.11]	2.12	3.12	[-4.00, 8.24]
*All other ∂*	1.8	0.57	[0.69, 2.91]***	1.72	4.54	[-7.19, 10.64]	2.54	2.05	[-1.48, 6.55]	2.28	2.27	[-2.17, 6.73]

Significant codes: 0.01 ‘***’ 0.05 ‘**’

*aβ- adjusted estimates

Se [β]- standard error of the estimates

DBP- diastolic blood pressure

SBP: systolic blood pressure

ℸ All other medications (see Table D **in S1 Text**)

∂ All other diagnoses (see Table B **in S1 Text**)

## Discussion

In the current study of Psychiatric Outpatients in Mozambique, we found major gaps in adherence to essential psychiatric medication. Only 7% of patients maintained adherence throughout the study follow-up period, with the median duration of adherence being just 60 days following the initiation of psychiatric medication. This adherence rate is lower compared to a 2020 global meta-analysis, which found that 48% of patients in Africa with major psychiatric disorders did not adhere to their psychotropic medication regimen [28].

Despite the overall high rates of nonadherence, our study identified specific sub-groups at an even higher hazard of medication noncompliance. Particularly, patients over 56 years of age exhibited a 30% higher hazard of medication non-adherence compared to those aged 18–35. This finding aligns with a study by Varghese et al., which reported that adverse drug effects are higher in older adults due to metabolic changes and decreased drug clearance that come with age. This risk is further compounded by the increasing number of drugs used in this population [29]. These findings are consistent with findings from the study by Abdulbari et al., who reported that patients aged 21–30 years were significantly more compliant with drug treatment than those over 30 years old [30]. In contrast, a study in Qatar examining medication compliance among patients with psychiatric disorders suggested that younger age might be a risk factor for medication non-adherence. This indicates that older individuals could adhere better to medication regimens due to a better understanding of their illness, its progression, and their prior treatment experiences [31]. In the Mozambican and LMIC contexts, further research is necessary to examine potential differential barriers to medication adherence by age. For instance, older individuals might face more challenges traveling long distances to clinics for regular medication, may struggle with physical limitations or comorbidities that hinder their ability to access care, may have less access to transportation, and might benefit from additional medication adherence support, such as community health worker visits or mobile health interventions.

In our study patients prescribed Amitriptyline had a 50% higher hazard of non-adherence compared to those prescribed Carbamazepine. In our cohort, 116 patients (15% of the sample) were prescribed Amitriptyline as their primary medication. Among these, 25% (n = 29) were diagnosed with nonorganic sleep disorders, 22% (n = 26) with depressive episodes, 16% (n = 9) with bipolar affective disorder, and 6% (n = 7) with epilepsy, among other conditions. Previous research has shown that patients taking older-generation tricyclic antidepressants, including Amitriptyline, often experience significant side effects such as insomnia, anxiety, dry mouth, and weight gain [32–34]. Potential causes of non-adherence may include the prevalence of negative side effects associated with tricyclic antidepressants, such as a global state of lethargy and demotivation, which significantly impact patients’ quality of life. While these medications are perceived to have beneficial effects on symptoms and relapse risk, many patients view them as the "least worst option," weighing the benefits against prominent adverse effects [35]. Additionally, a general shortage of psychotropic medications and disproportionate stockouts of amitriptyline may exacerbate the issue. The nature of mood disorders can make it harder to maintain a consistent routine of traveling to the health facility for follow-up visits or pickups [36] especially when patients feel their choices about medication are limited due to their illness or external pressures [35].

There is an increased need for a variety of medications to treat sleep disorders, depressive episodes, and bipolar disorder in Mozambique and other similar LMICs. Relying solely on older-generation tricyclics, which may have more side effects and lower efficacy, is insufficient. Patients often report feeling that their prescribing psychiatrists do not sufficiently acknowledge the negative impact of medication on their quality of life and physical health [37]. This lack of acknowledgment can lead to feelings of powerlessness, as patients perceive they have limited influence over decisions related to their medication [28]. To address this issue, several potential solutions could be considered. These include offering more flexible follow-up visit schedules, incorporating telehealth options, providing longer medication supplies between follow-up visits, and allowing patients to fill prescriptions at pharmacies closer to their homes. Furthermore, improving patient-provider communication, particularly in addressing concerns about medication side effects, may help to alleviate these feelings of disempowerment and ultimately improve medication adherence rates. These strategies could contribute to more patient-centered care and enhance long-term treatment outcomes.

In our examination of the effects of treatment duration, primary medications, primary diagnoses, and sociodemographic factors on disability scores, we found that even with challenges related to non-adherence to medication, each 30 days in mental health treatment resulted in meaningful and statistically significant lower disability scores over time. We also found that schizophrenia patients had higher disability scores than patients diagnosed with epilepsy. This difference might be attributed to the more chronic and debilitating nature of schizophrenia, which often requires more complex and sustained interventions. No association was found between disability scores and gender, age, marital status, or primary medication. Future research should explore the mechanisms behind the reduction in disability scores and test strategies to promote adherence and maximize gains in activities of daily living. Additionally, research should investigate if improvements are sustained and for how long.

Patients in the ’all other medication’ group had a weight that was 3.21 (95% CI:-6.41, 0.01) kg lower compared to those taking carbamazepine. We also found that males had lower diastolic blood pressure (4.39 mmHg; 95% CI: -8.51, -0.27) compared to females of similar age, medication, and diagnosis. These results underscore the importance of personalized treatment plans that account for specific diagnoses and individual patient characteristics. The observed weight differences suggest that medication choice can have significant effects on physical health, which should be considered when prescribing treatments.

The SMILES trial, a randomized controlled study of 67 adults with major depressive disorder, found that dietary improvement significantly reduced depressive symptoms and increased remission rates in moderate to severe cases. This suggests that dietary interventions can be a valuable adjunctive treatment for major depression [38]. Therefore, healthcare workers should enhance medication adherence with tailored interventions and consider integrating dietary and lifestyle modifications, especially for patients on medications with significant side effects or in populations at higher risk of non-adherence, such as in Mozambique.

This study has several limitations. First, the assessment of treatment adherence relied heavily on medication supply, which does not necessarily reflect patient non-compliance. The elevated non-adherence rate observed may be due to our method of measuring adherence time based on medication supply duration. This approach may be particularly unreliable in our context, as the reasons for the prescribed duration of medication were not always clear. While many studies have used medication duration as a proxy for adherence time [39]. Medication supply might be limited due to decisions by physicians to discontinue the treatment or prescribe medications for short durations because of supply shortages or clinical discretion, rather than due to patient non-compliance.

Furthermore, logistical challenges in LMICs exacerbate this issue, as patients often need to return to clinics for medication refills within 30 to 60 days, which can be time-consuming and costly compared to simply picking up prescriptions at local pharmacies, as is common in high-income countries. Additionally, the use of the ICD-10 classification system for primary diagnosis data may not be entirely accurate or appropriate for the setting [21]. Another significant limitation is the reliance on self-reported data to determine if patients took their medication as prescribed, which cannot be independently verified and may introduce bias. Moreover, since the data were collected from a limited number of hospitals in Mozambique, caution is advised when interpreting the results and making broader inferences about diagnostic and treatment patterns across the country. Lastly, our study utilized quantitative methods, which lacked direct patient involvement and limited our understanding of patients’ perspectives on medication adherence and treatment choices. Therefore, future research should consider employing qualitative designs to engage patients directly, providing richer insights into the subjective factors influencing adherence and enabling the development of interventions that are more aligned with patient needs and preferences.

## Conclusions

This study is the first to utilize a comprehensive dataset from eight clinics in Mozambique, employing a longitudinal approach over a two-year period to investigate medication non-adherence among Psychiatric Outpatients. It examined the relationships between non-adherence and clinical variables such as primary diagnosis, medication type, and sociodemographic factors. Importantly, it also explored how the primary medications prescribed impact changes in WHODAS scores, weight, and blood pressure over time. Our research found a very low adherence rate, with over 90% of patients failing to consistently follow their prescribed regimen. The median survival time prior to nonadherence among our study population was 60 days, after adjusting for facility-based variability. Patients prescribed amitriptyline and older patients are at a higher risk of non-adherence. The study also highlights significant associations between psychiatric diagnoses and a difference in WHODAS scores. These findings underscore the urgent need for targeted research into adherence support strategies to address the widespread challenge of medication non-adherence in Mozambique and potentially extend these strategies across sub-Saharan Africa.

## Supporting information

S1 Text Table A **in S1**: List of primary diagnoses group among Psychiatric Outpatients in Mozambique (February 2022—January 2024). Table B **in S1**: List of all other primary diagnoses group among Psychiatric Outpatients in Mozambique (February 2022—January 2024). Table C **in S1**: List of primary medication groups among Psychiatric Outpatients in Mozambique (February 2022—January 2024). Table D **in S1**: List of all other primary medication groups among Psychiatric Outpatients in Mozambique (February 2022—January 2024). Table E **in S1**: Test of proportional hazard assumption. Table F **in S1**: Factors Associated with Medication Non-Adherence Among Psychiatric Outpatients in Sofala and Manica Provinces, Mozambique (February 2022 –January 2024)–Pre-Diagnostic Test of Proportional Hazards Assumption. Table G **in S1**: Distribution of Primary Medications Across Different Primary Diagnoses Among Psychiatric Outpatients in Mozambique (February 2022—January 20, 2024).
(DOCX)

S1 Data Data. (XLSX)
