# Supplementary material for: Socio-demographic and clinical predictors of medication adherence among psychiatric outpatients in Mozambique: A two-year longitudinal survival analysis
Source: PLOS Ment Health. 2025 Feb 3;2(2):e0000236. doi: 10.1371/journal.pmen.0000236 (PMC12798487; doi:10.1371/journal.pmen.0000236)
Supplement: S1 Text — Table A in S1: List of primary diagnoses group among Psychiatric Outpatients in Mozambique (February 2022—January 2024). Table B in S1: List of all other primary diagnoses group among Psychiatric Outpatients in Mozambique (February 2022—January 2024). Table C in S1: List of primary medication groups among Psychiatric Outpatients in Mozambique (February 2022—January 2024). Table D in S1: List of all other primary medication groups among Psychiatric Outpatients in Mozambique (February 2022—January 2024). Table E in S1: Test of proportional hazard assumption. Table F in S1: Factors Associated with Medication Non-Adherence Among Psychiatric Outpatients in Sofala and Manica Provinces, Mozambique (February 2022 –January 2024)–Pre-Diagnostic Test of Proportional Hazards Assumption. Table G in S1: Distribution of Primary Medications Across Different Primary Diagnoses Among Psychiatric Outpatients in Mozambique (February 2022—January 20, 2024). (DOCX) [file pmen.0000236.s001.docx]

S1 Table A: List of primary diagnosis groups among Psychiatric Outpatients in Mozambique (February 2022 - January 2024)

| Primary diagnosis groups | N = 803 |
| --- | --- |
| Epilepsy | 406(50.5%) |
| Schizophrenia | 110(13.6%) |
| Delusional disorder | 47(5.8%) |
| Depressive disorder | 31(3.8%) |
| Mental and behavioral disorders due to drug and alcohol use | 51(6.4%) |
| All other diagnoses * | 141(17.6%) |
| Missing | 17(2.1%) |
| Total | 803(100%) |

S1 Table B: List of all other primary diagnosis groups among Psychiatric Outpatients in Mozambique (February 2022 - January 2024)

| All other primary diagnosis* | n=141 |
| --- | --- |
| Nonorganic sleep disorders | 29(21%) |
| Bipolar affective disorder | 26(18%) |
| Dementia | 22(16%) |
| Phobic anxiety disorders | 13(9%) |
| Nonorganic insomnia | 8(6%) |
| Acute stress reaction | 7(5%) |
| Mild intellectual disability | 6(6%) |
| Sexual dysfunction not caused by organic disorder | 4(3%) |
| Nonorganic enuresis | 4(3%) |
| Migraine syndromes | 3(2%) |
| Somatoform disorders | 2(1%) |
| Paranoid personality disorder | 2(1%) |
| Anorexia nervosa | 2(1%) |
| Trigeminal neuralgia | 1(1%) |
| Specific developmental disorders of scholastic skills | 1(1%) |
| Sexual desire disorders | 1(1%) |
| Schizotypal disorder | 1(1%) |
| Other behavioral and emotional disorders | 1(1%) |
| Organic amnestic syndrome | 1(1%) |
| Non-Organic hypersomnia | 1(1%) |
| Neurasthenia | 1(1%) |
| Mood [affective] disorders | 1(1%) |
| Mental and behavioral disorders associated with the puerperium | 1(1%) |
| Insomnia | 1(1%) |
| Hyperkinetic disorders | 1(1%) |
| Eating disorders | 1(1%) |
| Dissociative disorders | 1(1%) |
| Missing | 20(14%) |
| Total | 141(100%) |

S1 Table C: List of primary medication groups among Psychiatric Outpatients in Mozambique (February 2022 - January 2024)

| Primary medication groups | N = 803 |
| --- | --- |
| Carbamazepine | 398 (49.5%) |
| Amitriptyline | 116(14.4%) |
| Haloperidol | 86(10.7%) |
| Thioridazine | 64(7.9%) |
| All other primary medication ** | 120(14.9%) |
| Missing | 19(2.3%) |
| Total | 803(100%) |

S1 Table D: List of all other primary medication groups among Psychiatric Outpatients in Mozambique (February 2022 - January 2024)

| All other primary medication ** | n = 120 |
| --- | --- |
| Sodium valproate | 29(26%) |
| Chlorpromazine | 25(20%) |
| Fluphenazine | 16(13%) |
| Biperiden | 10(8%) |
| Imipramine | 7(6%) |
| Phenytoin | 2(2%) |
| Clonazepam | 2(2%) |
| Chlordiazepoxide | 2(2%) |
| Trifluoperazine | 1(1%) |
| Tramadol capsules | 1(1%) |
| Phenobarbital | 1(1%) |
| B complex | 1(1%) |
| Missing | 23(17%) |
| Total | 120(100%) |

S1 Table E: Test of proportional hazard assumption

| **Variable** | **Chi-square** | **Degree of Freedom** | **P-value** |
| --- | --- | --- | --- |
| Primary Medication | 4.86 | 4 | 0.3023 |
| Primary Diagnose | 16.81 | 5 | 0.0049 |
| Marital status | 3.44 | 5 | 0.6317 |
| Age | 3.31 | 5 | 0.3461 |
| Sex | 0.44 | 1 | 0.5094 |
| **Global** | **28.86** | **18** | **0.0501** |

S1 Table F: Factors Associated with Medication Non-Adherence Among Psychiatric Outpatients in Sofala and Manica Provinces, Mozambique (February 2022 – January 2024) **– Pre-Diagnostic Test of Proportional Hazards Assumption**

| Variables | Nonadherence  N (%) | Crude model | | Adjusted model | |
| --- | --- | --- | --- | --- | --- |
|  |  | HR | 95% CI | aHR* | 95% CI |
| Sex: Male | 383 (93%) | 0.96 | [0.83, 1.13] | 0.97 | [0.80, 1.19] |
| **Age (Years)** |  |  |  |  |  |
| *18-35 (ref)* | 434 (92%) |  |  |  |  |
| *<18* | 74 (96%) | 1.20 | [0.83, 1.73] | 1.26 | [0.83, 1.92] |
| *36-55* | 153 (92%) | 1.18 | [1.04, 1.34]** | 1.13 | [0.92, 1.40] |
| *56+* | 68 (94%) | 1.20 | [0.84, 1.70] | 1.31 | [1.10, 1.57]** |
| **Marital Status** |  |  |  |  |  |
| *Single (ref)* | 461 (93%) |  |  |  |  |
| *Married* | 55 (92%) | 1.05 | [0.77, 1.45] | 1.03 | [0.68, 1.58] |
| *Separated* | 26 (100%) | 1.00 | [0.52, 1.91] | 0.94 | [0.52, 1.70] |
| *Divorced* | 5 (83%) | 1.17 | [0.34, 3.95] | 0.72 | [0.19, 2.77] |
| *Widowed* | 40 (91%) | 0.94 | [0.70, 1.25] | 0.73 | [0.51, 1.04] |
| *Common Law* | 139 (935) | 1.01 | [0.87, 1.17] | 0.92 | [0.77, 1.12] |
| **Primary medication** |  |  |  |  |  |
| *Carbamazepine (ref)* | 366 (92%) |  |  |  |  |
| *Haloperidol* | 83 (97%) | 1.37 | [0.85, 2.20] | 1.38 | [ 0.92, 2.07] |
| *Amitriptyline* | 108 (93%) | 1.70 | [1.28, 2.26]*** | 1.54 | [1.19, 1.99]** |
| *Thioridazine* | 61 (95%) | 1.01 | [0.73, 1.41] | 0.95 | [0.59, 1.55] |
| *All other medications ℸ* | 109 (91%) | 1.21 | [1.10, 1.33]*** | 1.19 | [1.01, 1.41]** |
| **Primary Diagnosis** |  |  |  |  |  |
| *Epilepsy (ref)* | 372 (92%) |  |  |  |  |
| *Schizophrenia related disorders* | 206 (96%) | 1.07 | [0.94, 1.22] | 0.98 | [ 0.76, 1.25] |
| *Other psychotic and delusional disorders* | 43 (91%) | 1.23 | [0.90, 1.69] | 1.17 | [0.74, 1.83] |
| *Depression related disorders* | 30 (97%) | 1.54 | [1.28, 1.85]*** | 1.09 | [ 0.84, 1.41] |
| *Mental and Behavioral disorders due to substance use* | 48 (94%) | 1.33 | [0.77, 2.30] | 1.11 | [ 0.58, 2.14] |
| *All other “rare” diagnoses ∂* | 130 (92%) | 1.51 | [1.22, 1.87]*** | 1.20 | [ 1.02, 1.41]** |

*Significant codes: 0.01 ‘***’ 0.05 ‘**’*

** aHR - adjusted hazard ratio*

*ℸ All other medications with <30 patients grouped together (sodium valproate, chlorpromazine, fluphenazine, biperiden, imipramine, phenytoin, clonazepam, chlordiazepoxide, trifluoperazine, tramadol capsules, phenobarbital, and B complex)*

*∂ All diagnoses with < 30 patients grouped together (Nonorganic sleep disorders, bipolar affective disorder, dementia, phobic anxiety disorders, nonorganic insomnia, acute stress reaction, mild intellectual disability, sexual dysfunction not caused by organic disorder, nonorganic enuresis, migraine syndromes, somatoform disorders, paranoid personality disorder, anorexia nervosa, trigeminal neuralgia, specific developmental disorders of scholastic skills, sexual desire disorders, schizotypal disorder, other behavioral and emotional disorders, organic amnestic syndrome, non-organic hypersomnia, neurasthenia, mood [affective] disorders, mental and behavioral disorders associated with the puerperium, insomnia, hyperkinetic disorders, eating disorders, and dissociative disorders)*

S1 Table G: Distribution of Primary Medications Across Different Primary Diagnoses Among Psychiatric Outpatients in Mozambique (February 2022 - January 20, 2024)

| Primary medication | Primary diagnosis | | | | | | |  |
| --- | --- | --- | --- | --- | --- | --- | --- | --- |
|  | Epilepsy | Schizophrenia related disorders | Depression related disorders | Other psychotic and delusional disorders | Mental and behavioral disorder due to substance use | All others | Grand Total |  |
| Carbamazepine | 358 (90%) | 7 (2%) | 1(0%) | 6(2%) | 2(1%) | 24(6%) | 398 |  |
| Amitriptyline | 7 (6%) | 1 (1%) | 26(22%) | 2(2%) | 6(5%) | 74(64%) | 116 | |
| Haloperidol | 1 (1%) | 53 (49%) | 1(1%) | 12 (11%) | 25 (23%) | 17 (16%) | 109 |  |
| Thioridazine |  | 34 (53%) |  | 11 (17%) | 10 (16%) | 9 (14%) | 64 |  |
| All other medications | 39 (40%) | 15(15%) | 3(3%) | 16 (16%) | 9 (9%) | 15 (15%) | 97 |  |
| Grand Total | 405 (52%) | 110(14%) | 31(4%) | 47 (6%) | 52 (7%) | 139 (18%) | 784 |  |
